# The ProBio trial: molecular biomarkers for advancing personalized treatment decision in patients with metastatic castration-resistant prostate cancer

**DOI:** 10.1186/s13063-020-04515-8

**Published:** 2020-06-26

**Authors:** Alessio Crippa, Bram De Laere, Andrea Discacciati, Berit Larsson, Jason T. Connor, Erin E. Gabriel, Camilla Thellenberg, Elin Jänes, Gunilla Enblad, Anders Ullen, Marie Hjälm-Eriksson, Jan Oldenburg, Piet Ost, Johan Lindberg, Martin Eklund, Henrik Grönberg

**Affiliations:** 1 Department of Medical Epidemiology and Biostatistics, Karolinska Institutet, Stockholm, Sweden; 2 Department of Human Structure and Repair, Ghent University, Ghent, Belgium; 3 University of Central Florida College of Medicine, Orlando, FL USA; 4 Confluence Stat LLC, Orlando, FL USA; 5 Department of Radiation Sciences and Oncology, Umeå University, Umeå, Sweden; 6 Länssjukhuset Sundsvall Härnösand, Sundsvall, Sweden; 7 Department of Immunology, Genetics and Pathology, Uppsala Universitet, Uppsala, Sweden; 8 Department of Oncology-Pathology, Karolinska Institutet, Stockholm, Sweden; 9 Capio St. Görans Hospital, Stockholm, Sweden; 10 Division of Medicine, University of Oslo, Oslo, Norway; 11 Department of Radiotherapy and Experimental Cancer Research, Ghent University, Ghent, Belgium

**Keywords:** Clinical trial platform, Genetic biomarker, Prostate cancer, Precision medicine

## Abstract

**Background:**

Multiple therapies exist for patients with metastatic castration-resistant prostate cancer (mCRPC). However, their improvement on progression-free survival (PFS) remains modest, potentially explained by tumor molecular heterogeneity. Several prognostic molecular biomarkers have been identified for mCRPC that may have predictive potential to guide treatment selection and prolong PFS. We designed a platform trial to test this hypothesis.

**Methods:**

The Prostate-Biomarker (ProBio) study is a multi-center, outcome-adaptive, multi-arm, biomarker-driven platform trial for tailoring treatment decisions for men with mCRPC. Treatment decisions in the experimental arms are based on biomarker signatures defined as mutations in certain genes/pathways suggested in the scientific literature to be important for treatment response in mCRPC. The biomarker signatures are determined by targeted sequencing of circulating tumor and germline DNA using a panel specifically designed for mCRPC.

**Discussion:**

Patients are stratified based on the sequencing results and randomized to either current clinical practice (control), where the treating physician decides treatment, or to molecularly driven treatment selection based on the biomarker profile. Outcome-adaptive randomization is implemented to early identify promising treatments for a biomarker signature. Biomarker signature-treatment combinations graduate from the platform when they demonstrate 85% probability of improving PFS compared to the control arm. Graduated combinations are further evaluated in a seamless confirmatory trial with fixed randomization. The platform design allows for new drugs and biomarkers to be introduced in the study.

**Conclusions:**

The ProBio design allows promising treatment-biomarker combinations to quickly graduate from the platform and be confirmed for rapid implementation in clinical care.

**Trial registration:**

ClinicalTrials.gov Identifier NCT03903835. Date of registration: April 4, 2019. Status: Recruiting.

## Introduction

Despite multiple therapeutic avenues for metastatic castration-resistant prostate (mCRPC), their impact on prolonging survival remains modest [1]. The wide range of clinical outcomes and the plethora of potential resistance mechanisms for each treatment suggest that an ideal therapeutic approach requires accurate patient selection taking tumor biology into account [2, 3]. At present, no clear guidelines exist on how to choose the right treatment for the right patient at the right time. With multiple alternative therapies and a number of new therapies expected to be approved for patients with mCRPC, it is imperative to optimize the treatment selection and identify the optimal sequencing of available therapies [1].

Here, we describe the design of the ProBio study, a prospective multi-center platform trial for tailoring treatment decision-making for men with mCRPC. ProBio is an outcome adaptive, multi-arm, biomarker-driven platform trial with the aim of prospectively identifying and validating predictive molecular biomarkers. Specifically, we will test whether somatic and germline alterations can predict if a patient is more likely to benefit from receiving a particular therapy [4, 5]. The adaptive design is particularly suitable for addressing multiple questions at once and allows for promising treatment-biomarker signatures to quickly graduate from the trial and to be faster implemented in routine clinical care [6, 7]. Treatment-biomarker combinations may also exit the trial if there is accumulated evidence of them being ineffective. The platform design allows for new drugs and new biomarkers to be introduced in ProBio, to enrich the options for guiding treatment selection for men with mCRPC.

### Background and rationale

Prostate cancer is the most common cancer and the second leading cause of cancer-related death among men in the Western world [8]. mCRPC is a lethal form of advanced or metastatic prostate cancer, characterized by progressive disease under androgen deprivation [9]. Currently, the most common systemic standard-of-care (SOC) therapies for these patients are second-generation hormonal therapy (abiraterone acetate and enzalutamide), chemotherapy (docetaxel, cabazitaxel), and radionuclide therapy (radium-223) [10]. Novel targeted agents (e.g., PARP and PD-1 inhibitors) are expected to soon enrich the landscape of available treatments for mCRPC patients [1, 3].

The number of therapeutic options for mCRPC patients is increasing, but the response rates in unselected patient populations remain moderate. This leads to missed opportunities of immediately selecting optimal therapy, unnecessary side-effects for the patient, and costs to the health care systems. Although approved for unselected mCRPC patients, these SOC agents are likely more beneficial for particular subgroups of the patient population [3]. Biomarker-driven clinical trials for mCRPC have been hampered by the difficulty of obtaining metastatic tissue [11]. Also, profiling a single metastatic lesion is not capable of providing the full spectrum of the molecular heterogeneity that may exist within the patient [12, 13]. A liquid biopsy, either in the form of circulating tumor cells (CTCs) or tumor-derived cell-free DNA (circulating tumor DNA, ctDNA), is an attractive alternative [14, 15]. Circulating tumor DNA has been shown to be highly concordant to metastatic tissue for detecting somatic variations and allows for longitudinal monitoring and detection of acquired resistance [16–19]. The use of molecular biomarkers has been successful for patient prognostication and holds the promise to inform treatment selection as predictive biomarkers for mCRPC [20]. Currently, the IND.234 trial is applying ctDNA sequencing in second- or third-line mCRPC to test pre-defined biomarker-treatment hypothesis for enriched responses that may subsequently be investigated in randomized trials [21].

The multiplicity of available treatments with an evolving therapeutic landscape and the molecular heterogeneity with low prevalence of patients carrying a specific marker highlights the limitations of current clinical trials in evaluating the efficacy of comparative treatments and potentially treatment-predictive biomarkers [3]. A possible remedy for addressing multiple research questions within the same clinical trial is the implementation of a platform design [22–24]. The multi-arm structure of a platform trial allows to compare alternative therapies with a common control group. Given the flexibility of a platform design, it is possible to add or drop experimental arms and use the accumulated data to change the course of the trial according to prespecified criteria. The multiplicity of available therapies under investigation within a heterogeneous patient population characterized by biomarkers leads to large number of testable hypotheses in the trial. The outcome-adaptive component of ProBio can assign more patients to promising arms, thus allocating the available patients to test the most plausible hypotheses (conditional on the data collected within the trial). In addition, it can be argued that it is also more beneficial for the participants in the study (since patients on average have higher probability to be assigned to effective treatments), and that it can reduce costs [6].

### The ProBio trial

**Fig. 1:**
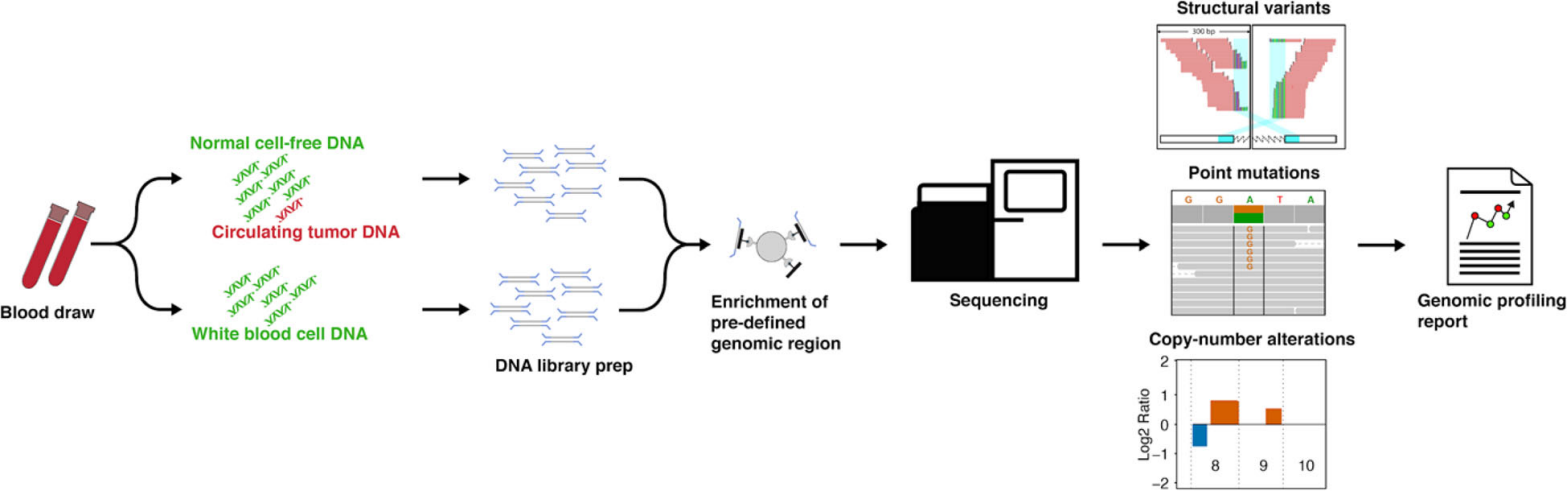
Genomic profiling in the ProBio platform trial. Two 10-ml tubes of blood are drawn from each study participant and plasma is enriched. Extraction is performed to obtain cell-free DNA from plasma and germline DNA from white blood cells. Targeted sequencing is applied on both cell-free and germline DNA using the ProBio-panel. The ProBio panel covers mutations in 78 genes, structural variants in 11 genes and allows for interrogation of genome-wide copy-number alterations, microsatellite instability, and hypermutation. Sequence data is processed using an in-house developed bioinformatics infrastructure (https://autoseq-docs.readthedocs.io). All variants are manually examined to remove false positive calls. This information is condensed into a report which contains the biomarker profile for subsequent randomization of the study participants. Study participants that progress are reanalyzed and re-randomized

**Fig. 2:**
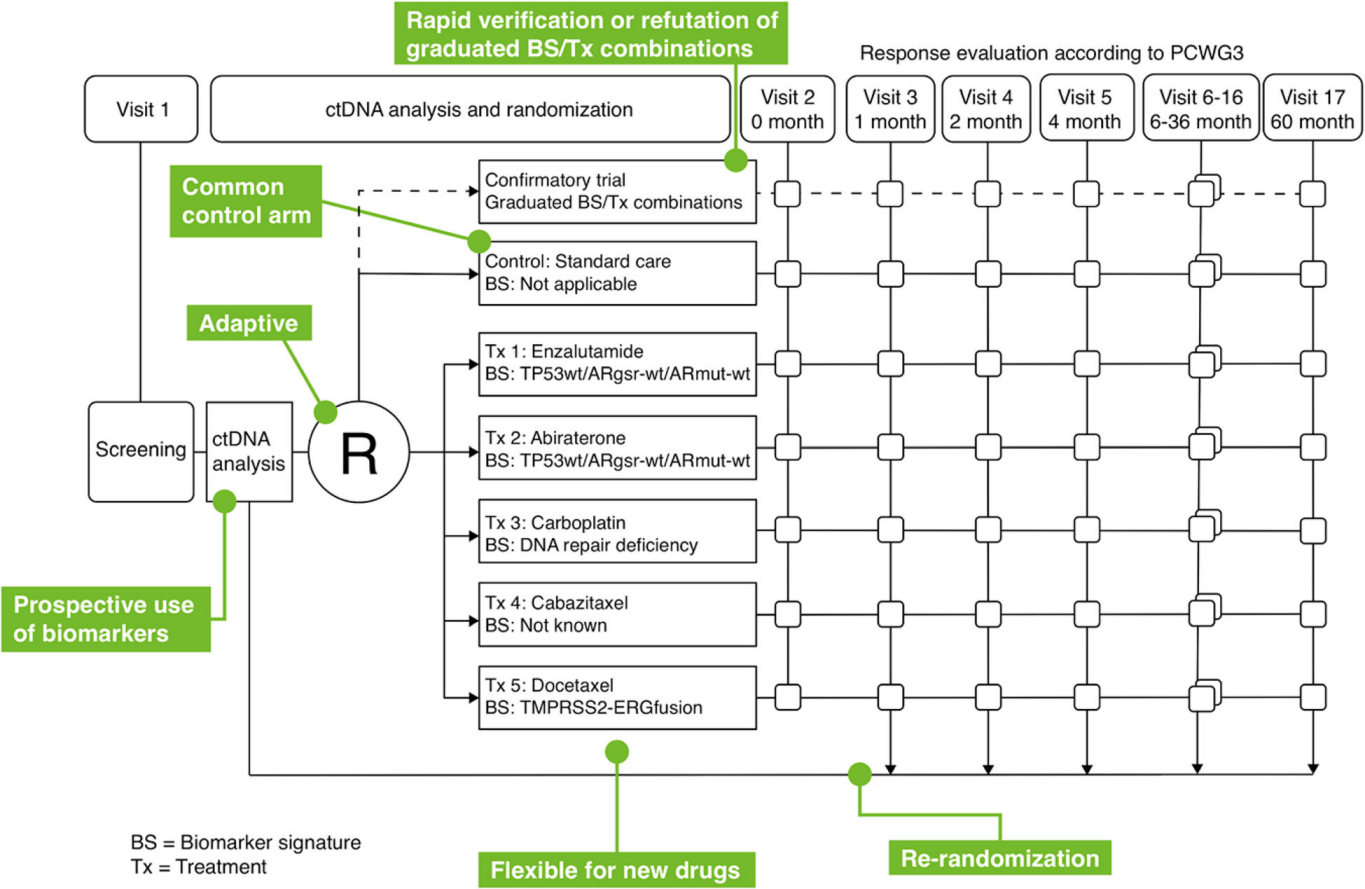
Study design of the ProBio platform trial. Participants who meet the inclusion criteria and agreed to participate in the study are genotyped and their biomarker profile is derived. Based on their biomarker subgroup combination they are randomized to either the control group (standard-of-care) or one of the active arms. Patients are regularly followed through the study. Their outcome data is used to adapt the randomization probabilities, assigning more patients to more beneficial therapies within a biomarker signature. Upon the first progression in the study, patients will be re-genotyped and re-randomized to an alternative arm based on their updated biomarker profile

**Fig. 3:**
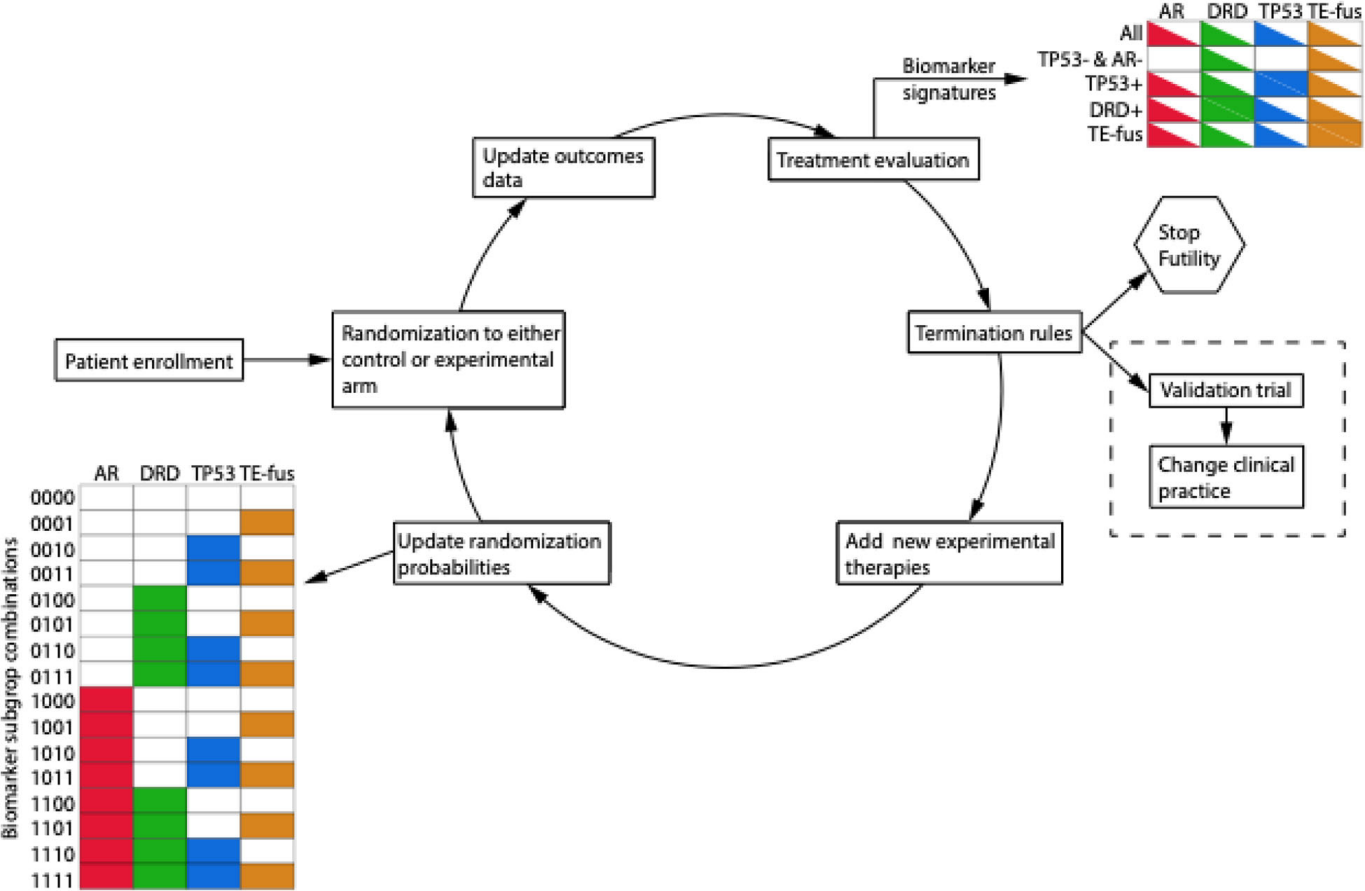
Life cycle of the ProBio platform trial. After written informed consent, the biomarker subgroup combination of the patient is determined and used for randomization to either the control group (standard of care) or one of the experimental arms. Outcome data are updated monthly throughout the trial and will be used to calculate the probabilities of superiority for the active arms over the control group for each biomarker signature of interest. Based on the selected threshold, a decision to continue enrollment or to terminate (for futility or superiority) each treatment-biomarker signature will be made. As treatment-biomarker signatures leave the platform, new treatments can possibly entry in the study. The outcome data is also used to update the randomization probabilities within the biomarker subgroup combinations. Graduating treatment-biomarker signatures will enter a confirmatory trial to validate the hypotheses generated from the platform

ProBio is the first biomarker-driven outcome-adaptive trial for mCRPC, designed to accelerate the implementation of novel results generated by molecular epidemiology into routine clinical care. ProBio incorporates several multidisciplinary innovations including prospective liquid biopsy-based molecular profiling (Fig. 1), novel features in the clinical study design (Figs. 2 and 3), and dedicated solutions for logistics and clinical implementation. The trial was initiated in Sweden and will expand internationally during 2020 to hasten recruitment of a large number of patients. ProBio will create a learning environment not only to identify biomarker profiles where therapies are more effective, but also to answer a multiplicity of prespecified research questions (e.g., surrogacy role of ctDNA fraction, identification of new biomarker signatures based on collected data, comparing RNA analysis from plasma and thrombocytes) and new hypotheses that will arise throughout the study.

### Trial design

ProBio is an outcome-adaptive, multi-arm, biomarker-driven platform trial to determine whether treatment selection based on a liquid biopsy-derived biomarker profile can prolong progression-free survival (PFS) in men with mCRPC. The trial will be analyzed within a Bayesian framework where alternative treatments will be compared within biomarker signatures in terms of their probability of superiority, using a common comparator [25].

Patients are stratified based on their ctDNA biomarker signature and randomized to either one of the experimental arms where treatment decisions (abiraterone, enzalutamide, carboplatin, docetaxel, or cabazitaxel) are based on the biomarker signatures or the control group defined by current clinical practice (Fig. 2). As the therapeutic landscape is quickly evolving, new drugs may be introduced in the active arms, upon protocol amendment. Radium-223 is not included due to the recent EMA recommendations restricting its use only to mCRPC patients who already received two treatments. Carboplatin is included despite the lack of an indication for prostate cancer since there is accumulating evidence that platinum-based chemotherapy is effective in tumors with defects in the DNA repair genes [26]. Several therapies will be available for treatment of patients in a biomarker signature, and—conversely—a given as well as one treatment can be administrated for patients with different biomarker signatures.

The outcome-adaptive randomization is implemented to assign more patients to biomarker-treatment combinations with the highest probability of being superior to SOC and to early identify drugs which are promising in the subpopulation of patients defined by a specific biomarker signature. As data accumulates and it begins to become evident which treatments are least effective for certain biomarker signature, fewer of those patients are randomized to poorly performing therapies. This has the important advantages of providing patients with a treatment more likely to work for them, rather than a less effective therapy, but also of using the finite resources (patients) in a more suitable way: assigning patients toward the latter stages of the trial only to the treatments still competing to be the best treatment for that disease type.

Biomarker-treatment combinations that exit the trial based on superiority are further evaluated in a seamless confirmatory trial nested in the ProBio platform using fixed randomization (Fig. 3). Upon progressive disease, the patient will re-enter the trial and be re-randomized one additional time (with a maximum of 2 randomizations) to another treatment based on their current biomarker profile. Patients that have undetectable ctDNA [27] and do not harbor any relevant gDNA alterations cannot be randomized and will enter an observation arm of the study where SOC is administered. Both the re-randomization and the observation arm will provide important insights for selecting an optimal treatment sequence for mCRPC patients.

### Inclusion/exclusion criteria

ProBio will enroll patients with mCRPC, aged 18 years and above, with an Eastern Cooperative Oncology Group (ECOG) performance status of 0–2, histologically confirmed prostate adenocarcinoma, and castrate levels (< 50 ng/dl) of serum testosterone, conforming to EAU guidelines [2]. The patient should have an adequate health, bone-marrow, hepatic and renal function to receive all available treatments in the trial. Distant metastatic disease needs to be documented by positive Tc-99 bone scintigraphy or by computed tomography (CT) or magnetic resonance imaging (MRI) scans. The ProBio trial will initially allow to recruit mCRPC patients starting both 1st- or 2nd-line systemic therapy for progressive disease, but will in the near future limit enrollment to 1st-line patients to infer a better understanding on treatment sequencing. Patients are not eligible if they have received more than two of the drugs under investigation in the platform, prior to study inclusion.

### Biomarker subgroup combinations and signatures

Molecular characterization of the tumor through a ctDNA-driven liquid biopsy is a key feature of the ProBio trial. Multiple molecular perturbations (splice variants, point mutations, amplifications, and genomic rearrangements) can be associated with treatment outcome and response for men with mCRPC [28–30]. In men treated with enzalutamide or abiraterone, the AR-V7 splice variant (up to 60% prevalence) has been suggested as a negative response marker [31, 32]. However, the combination of TP53 inactivation (occurring in 25–40% of mCRPC patients) and multiple AR alterations has demonstrated more promising results [33–35]. Metastatic prostate cancer with DNA repair deficiency (DRD), occurring in about 20% of mCRPC cases, has been suggested to have a higher sensitivity to PARP inhibition [36] and platinum-based chemotherapy [37, 38]. The FDA approved the anti-PD1 immunomodulator pembrolizumab in patients with any microsatellite instable (MSI) or mismatch repair deficient (dMMR) solid tumor [39, 40]. Approximately 3–4% of mCRPC are MSI positive [29, 41], with partial or complete responses to checkpoint inhibition being observed in up to 50% of these patients [40, 42–44]. Finally, The *TMPRSS2-ERG* gene-fusion, occurring in 40–50% of prostate cancer [34, 45], has been suggested to predict response to docetaxel [46].

The ProBio trial will initially evaluate four classes of pre-defined genomic biomarker signatures, which have been recognized as the major candidates for guiding prognosis and treatment decision [27, 29, 47]:

1. Mutations and structural rearrangements in *AR*;
2. Mutations, homozygous deletions, and structural rearrangements in *TP53*;
3. DNA-repair deficiency by detection of mutations, homozygous deletions, and structural rearrangements in ATR, ATM, BARD1, BRCA1, BRCA2, BRIP1, CHEK2, FANCA, MRE1, NBN, PALB2, RAD50, RAD51, RAD51B, RAD51C, and RAD51D; and
4. *TMPRSS2-ERG* fusions by structural rearrangements and deleterious events.

New biomarkers that will be proven relevant for treatment response of mCRPC patients may be prospectively introduced in ProBio. The combination of these 4 biomarkers defines the biomarker subgroup combination of a patient, i.e., the molecular characteristics of the tumor including germline DNA alterations (Fig. 3). Randomization to either the control group or one of the active treatments occurs conditional on the patient’s biomarker subgroup combination, where a patient belongs to one and only one subgroup. Initially, four binary biomarkers will be considered, which defines 2^4^ = 16 biomarker subgroup combinations. The effect of a treatment within one of these 16 biomarker subgroup combinations is however typically of limited interest because of the low prevalence of each combination. However, treatments may be more effective in a subpopulation defined by a group of biomarkers, all harboring alterations in, e.g., the target pathway that a specific drug aims to block. We refer to such groups as a *biomarker signature* [48]. Initially the ProBio trial will test 5 different biomarker signatures (Table 1). Contrary to the biomarker subgroup combinations, a patient may belong to more than one biomarker signature (Fig. 3). For example, an *AR* and *TP53* wild-type patient belongs both to the signature “all patients” and “*AR−* and *TP53−*”. While randomization happens at a biomarker subgroup combination level, therapies are evaluated at the higher level of biomarker signatures.

**Table 1:** Definition of the 5 candidate biomarker signatures and estimated prevalences with included biomarker subgroup combinations, in the order AR, DRD, TP53, and TEfus (where “+” indicates mutated, “−” wild-type/non-mutated)

Signatures	−	−−−+	−−+−	−−++	−+−−	−+−+	−++−	−+++	+−−−	+−−+	+−+−	+−++	++−−	++−+	+++−	Prev
All	x	x	x	x	x	x	x	x	x	x	x	x	x	x	x	1
TP53**−** and AR**−**	x	x			x	x										0.5
TP53+			x	x			x	x			x	x			x	0.37
DRD+					x	x	x	x					x	x	x	0.19
TEfus+		x		x		x		x		x		x		x		0.32

### Outcome adaptive randomization

The biomarker subgroup combination works as a stratification variable for randomization procedure. Once the unique patient’s biomarker subgroup combination has been identified, the patient is randomized to either the control or one of the active treatments based on prespecified randomization probabilities. The control group reflects current clinical practice, i.e., treatment selection according to national guidelines without the information on the tumor biology, and consists of a mix of available treatments. Given the stratified randomization, there will be a separate control group for all the biomarker subgroup combinations that will work as comparator for the active treatments within the biomarker subgroup combination or the biomarker signatures (Fig. 1).

Fixed randomization within biomarker subgroup combinations will be implemented before accruing a minimum number of patients across the total of the active arms (*n* = 50), after which the adaptation starts to be applied. Thenceforth, experimental therapies will be randomized proportional to their Bayesian probability of prolonging PFS compared to the control as a measure of how well a treatment is working. For each biomarker subgroup combination, we will assure that the control groups receive at least as many patients as any single drug in the experimental arm (i.e., mimicking 1:1 randomization between the control and the most promising treatment within the biomarker subgroup combination). Randomization probabilities will be updated monthly based on the accumulated data throughout the trial. We chose to update randomization probabilities using the observed PFS times because time to progression in first-line and all-comer mCRPC patients can be relatively short [28, 49].

### Evaluation of therapies

Therapies will be evaluated in two different stages. In the first one, all the therapies will be compared to the respective controls in all the biomarker signatures (screening stage). Treatments that show evidence of superiority in selected biomarker signatures will graduate from the screening stage and enter the confirmatory stage, where the therapies will be tested only for the associated graduating signature. As patients may enter the trial at different stages of the disease and progress, we will stratify the analyses by line of treatment from the development of mCRPC.

#### Screening stage

Therapies will be evaluated for effectiveness as compared to the control group separately for each biomarker signature of interest. The main outcome is PFS, where progression is defined according to Prostate Cancer Working Group 3 [50]. We will use Bayesian methods for survival analysis to contrast the distributions of PFS times across the active arms and within the biomarker signatures [25]. In particular, we will adopt the two-parameter Weibull distribution to model the observed PFS times. We chose the conjugate prior for the Gamma-Weibull model with hyperparameters alpha = 10 and beta = 80, which correspond approximately to the information from 10 patients. The conjugate prior facilitates Bayesian inference as posterior distributions can be computed without the need of implementing and tuning Markov chain Monte Carlo methods. The posterior distributions of the modeled parameters will be used to evaluate the effectiveness of the active arms in the trial by computing the probabilities of superiority within the biomarker signatures, i.e., the probability that each treatment offers a longer time to progression than the control in each biomarker signature. After enrolling a minimum number of 20 patients, an active treatment may graduate and exit from the platform trial for a specific biomarker signature if its probability of superiority exceeds a predefined threshold (85%). To avoid the problem of treatments only graduating in signatures with high prevalence, we also require that the graduating treatment is performing well in all the biomarker subgroup combinations which belong to the graduating signature. This is done by computing the probabilities of superiority within the biomarker subgroup combinations. If a treatment, instead, appears to be particularly ineffective (i.e., probability of superiority less than 15%), it will exit the trial for that biomarker signature. If none of the conditions are met and the maximum number of patients in the biomarker signature of interest is not reached (*n* = 150), randomization to the treatment under investigation will continue. Decisions about graduating and dropping treatments within biomarker signatures will be made by advice from experts in the data and safety monitoring board. Once a treatment graduates for a biomarker signature, it will exit the study and enter seamlessly in the confirmatory trial which is nested within the ProBio platform.

#### Confirmatory trial

The rationale for the ProBio platform study design is to learn from the data that accumulates in the trial and quickly generate solid hypotheses in a prospective way. To generate practice-changing level of evidence, we will subsequently validate the promising biomarker-therapy combinations in a confirmatory trial (Fig. 2). When a treatment graduates from the platform for a biomarker signature, it will no longer be available in the active arms of the associated signature and will enter in a side trial nested within the ProBio platform. The control group for the biomarker subgroup combinations belonging to the graduating signature will be divided in two halves using fixed randomization, the first receiving SOC and the other the graduating treatment. The only comparison will be made between the graduating treatment and the control group for the confirmatory trial (without using the controls in the ProBio platform). On the other hand, the patients in the SOC arm will at this stage also act as a comparator for the remaining active arms in the platform study. The confirmatory trial will be analyzed in a frequentist manner.

### Power and sample size considerations

We have selected the threshold values for graduation of a treatment-biomarker combination or stopping for futility based on extensive simulation studies, since operating characteristics cannot be easily calculated for complex platform trials [7]. The calibration of those thresholds has been performed to control the type-I error and assure an adequate power for graduating treatment-biomarker combinations.

In the simulations, we assumed multiple scenarios ranging from no differences in treatments in any of the biomarker combinations to treatments prolonging the mean PFS by 5 to 10 months. In terms of sample size, the average number of participants in a treatment-biomarker signature combination ranged from 70 to 95 to achieve graduation depending on the assumed scenario. The average time in which effective signature-treatment combinations remained in the trial ranged from 21 to 30 months. Given the multiplicity of therapies, we controlled the overall type-I error to be lower than 30% (10% for the individual drugs), with varying power figures up to 80% for graduating treatment-biomarker combinations. The choice of an adequate alpha level in the independent confirmatory trial will ensure an overall type-I error below 5% (such as alpha = 15%, overall type-I error = 15%·30% = 4.5%).

A comprehensive description of the simulation study will be published in a future manuscript, detailing the statistical aspects of the trial. A summary of the simulations’ results can be found in the protocol. A web interface to the simulations is available at http://alessiocrippa.com/shiny/probio_dsmb/.

### Final and trajectory analysis of the screening stage

The main and final analysis of all the active arms will be performed at the end of the screening stage of the ProBio trial. The treatment comparison, regardless if it has been validated or not, will be based on PFS within a biomarker signature. The measure of effectiveness will be the probability of superiority over the control group computed using parametric models within a Bayesian framework. Additional endpoints, such as response rates, overall survival, quality-of-life measures, toxicity, and health economy, are also of interest. Depending on the nature of the secondary endpoint, we will contrast their distributions in the active treatments versus the control using relevant Bayesian models.

The aim of ProBio is to assess treatment allocation based on molecular profiling compared to SOC. That is, we want to test whether molecular profiling leads to better treatment selection than if the treating physicians make the choices. This means that the control group is a mix of treatments (some present also in the active arms). As a secondary aim of ProBio, we will also compare the “efficacy” of different treatments within specific signatures. This aim addresses whether a given treatment works better in specific signatures than other treatments.

In the analysis of the sequences of treatments, we will contrast the patients’ trajectories in terms of their overall time since randomization in ProBio, given by the sum of the PFS times under the two possible consecutive randomizations. The comparison will be performed using Bayesian mixed-effects model with an interaction between time and treatment arm (active versus control), to test differences in the progression of the disease. The dependence between repeated observations will be taken into account by the random-effects in the hierarchical model.

### Patients’ pathways

ProBio patients can follow different pathways within the trial depending not only on their biomarker subgroup combinations, but also from their clinical features and different timing of randomizations among others. We have provided an overview of the most relevant pathways for a ProBio patient in Supplementary Figure 1.

New patients may end up in an observational arm if their biomarker subgroup combination cannot be inferred (undetectable ctDNA, technical failure, or microsatellite instability). Otherwise, patients will be randomized either to the control group (SOC) or one of the active treatments. After progressive disease, patients may be rerandomized to another active treatment, but patients in the control group and in the observational arm will remain in their arm upon progression and will keep receiving SOC. As patients might be unfit or unwilling to continue the trial, both the patient and treating physician might choose to discontinue the patient and exit the trial. In the later stage of the trial, if the biomarker subgroup combination of a new patient belongs to one of the graduating biomarker signatures, the patient will enter the confirmatory trial with fixed randomization to the control (SOC) or the graduating active treatment. Finally, upon progressive disease after the second randomization, all patients will discontinue and exit the ProBio trial.

### Current status of the ProBio trial

The ProBio trial is currently opening up at multiple healthcare centers across Sweden (5 sites were opened during the Spring of 2019 and an additional 6 sites will open during 2020) with an expansion to other Scandinavian countries and Belgium planned for 2020. So far, 53 patients have been enrolled in the study, and the accrual rate is currently reaching about seven patients per month. Biomarker signatures could be inferred in 37/58 enrolled patients with a median turnaround time of 15 days (from blood collection to reporting randomization results). In 5 cases, the ctDNA fraction was below 1%, resulting in an incomplete assessment of the somatic biomarker signature status. These patients entered an observational standard-of-care arm of the study and hence a potential randomization for their subsequent line of therapy.

The ProBio investigators are currently seeking to expand the described trial design toward earlier stages of the disease. This will be increasingly important in the context of the changing treatment landscape of metastatic hormone-sensitive prostate cancer, where the introduction of chemohormonal therapy (GETUG-15, CHAARTED, STAMPEDE), upfront association of abiraterone (LATITUDE, STAMPEDE), enzalutamide (ENZAMET), or apalutamide (TITAN) with standard ADT with or without local therapy of the prostate in case of de novo M1 disease (STAMPEDE), will demand a similar model for improving treatment selection for patients with advanced cancer.

## Discussion

ProBio is an innovative platform design for enhancing treatment selection for mCRPC patients. Its design implements several innovations compared to standard clinical trials, including a flexible structure for adding or dropping treatments and/or biomarker signatures, adapting randomization probabilities based on the accrual data, addressing multiple hypotheses within the same study design, and validating promising therapies seamlessly to quickly change standard-of-care.

Adaptive platform trials have been advocated as an ideal solution for addressing multiple scientific questions at once [7], such as evaluating multiple treatments in a heterogeneous population. Outcome-adaptive randomization, which is a common feature of adaptive trials and employed in ProBio, has been criticized for bringing modest-to-no benefits to the operating characteristics of a trial [51, 52] and for being unethical [53] (Buyse et al. 2016). Multiple simulation studies have shown, however, that multi-arm designs employing adaptive outcome-randomization strategies that protect control allocation over time, such as the one used in ProBio, proved to be superior to designs using fixed randomization probabilities, including multi-arm multi-stage designs [54–56]. Lastly, while a discussion of the ethical objections to outcome-adaptive randomization is outside the scope of this paper, we note that strong counterarguments to those objections have been put forward [57, 58].

In conclusion, this platform design has the potential to quickly corroborate hypotheses and generate new evidence which would not have been otherwise possible in a conventional randomized trial. In addition, the ProBio trial may dramatically reduce the years and the costs associated with changing current clinical practice. Treatments that will graduate from the platform but fail in the confirmatory trial may still be valuable for refining biomarker signatures and inform treatment selection for patients outside the study. By allowing patient accrual and re-randomization throughout different lines of systemic therapy for mCRPC, ProBio will allow us to improve treatment selection that will maximize health outcomes for the patient, and will provide essential insights into optimal treatment sequencing regimen.

### Trial status

The trial protocol, version number 3.0, was approved in April 2019 and is ongoing. The recruitment of patients was started February 1, 2019, and will continue for at least 3 years.

## Supplementary information

**Additional file 1: Supplementary Figure 1**. Patients pathway. Patients in ProBio may end up in an observational arm where standard-of-care (SOC) is administered. This may have been the result of low or undetectable ctDNA (Pathway 1, 10 & 14), technical failure during liquid biopsy profiling (Pathway 2, 11 & 15) or identification of the MSI or hypermutator (Pathway 3). When biomarker signature can be inferred, the trajectory of the new patient depends whether particular biomarker signature-treatments combinations have graduated. If none are available, the patient will be randomized either to the control group (SOC) or one of the active treatments (Pathway 4 & 5). Upon first randomisation and progressive disease, the allocated patients remain in their respective arm for re-randomisation (Pathway 8 & 12). However, as patients might be unfit or unwilling to continue the trial after their first randomisation, both patient and treating physician might chose to discontinue the patient and exit the trial (Pathway 9 & 13). If a newly entered patient has a biomarker signature for which a graduated biomarker signature-therapy combination is available, the patient will enter a confirmatory trial pathway, which uses fixed randomisation between control and graduating treatment (Pathway 6-7). Finally, upon progressive disease after the second randomisation (Pathway 16 & 17), or after randomisation within the confirmatory trial testing the graduated biomarker signature-therapy combination (Pathway 18), all patients will discontinue and exit the ProBio trial.

## Data Availability

The questionnaires and coding sheets used in the study, as well as anonymized data, will be included in or as supplementary files to articles published, as required. Trial results will be disseminated to participants and relevant health-care providers via a stakeholder committee.

## Abbreviations

mCRPC: Metastatic castration-resistant prostate cancer
CT: Computed tomography
CTCs: Circulating tumor cells
ctDNA: Circulating tumor DNA
DRD: DNA repair deficiency
MRI: Magnetic resonance imaging
PFS: Progression-free survival
SOC: Standard of care
