# Supplementary material for: The ProBio trial: molecular biomarkers for advancing personalized treatment decision in patients with metastatic castration-resistant prostate cancer
Source: Trials. 2020 Jun 26;21:579. doi: 10.1186/s13063-020-04515-8 (PMC7318749; doi:10.1186/s13063-020-04515-8)
Supplement: Supplementary file 1 — Additional file 1: Supplementary Figure 1. Patients pathway. Patients in ProBio may end up in an observational arm where standard-of-care (SOC) is administered. This may have been the result of low or undetectable ctDNA (Pathway 1, 10 & 14), technical failure during liquid biopsy profiling (Pathway 2, 11 & 15) or identification of the MSI or hypermutator (Pathway 3). When biomarker signature can be inferred, the trajectory of the new patient depends whether particular biomarker signature-treatments combinations have graduated. If none are available, the patient will be randomized either to the control group (SOC) or one of the active treatments (Pathway 4 & 5). Upon first randomisation and progressive disease, the allocated patients remain in their respective arm for re-randomisation (Pathway 8 & 12). However, as patients might be unfit or unwilling to continue the trial after their first randomisation, both patient and treating physician might chose to discontinue the patient and exit the trial (Pathway 9 & 13). If a newly entered patient has a biomarker signature for which a graduated biomarker signature-therapy combination is available, the patient will enter a confirmatory trial pathway, which uses fixed randomisation between control and graduating treatment (Pathway 6-7). Finally, upon progressive disease after the second randomisation (Pathway 16 & 17), or after randomisation within the confirmatory trial testing the graduated biomarker signature-therapy combination (Pathway 18), all patients will discontinue and exit the ProBio trial. [file 13063_2020_4515_MOESM1_ESM.docx]

**Supplementary Figure 1: Patients pathway**. Patients in ProBio may end up in an observational arm where standard-of-care (SOC) is administered. This may have been the result of low or undetectable ctDNA (Pathway 1, 10 & 14), technical failure during liquid biopsy profiling (Pathway 2, 11 & 15) or identification of the MSI or hypermutator (Pathway 3). When biomarker signature can be inferred, the trajectory of the new patient depends whether particular biomarker signature-treatments combinations have graduated. If none are available, the patient will be randomized either to the control group (SOC) or one of the active treatments (Pathway 4 & 5). Upon first randomisation and progressive disease, the allocated patients remain in their respective arm for re-randomisation (Pathway 8 & 12). However, as patients might be unfit or unwilling to continue the trial after their first randomisation, both patient and treating physician might chose to discontinue the patient and exit the trial (Pathway 9 & 13). If a newly entered patient has a biomarker signature for which a graduated biomarker signature-therapy combination is available, the patient will enter a confirmatory trial pathway, which uses fixed randomisation between control and graduating treatment (Pathway 6-7). Finally, upon progressive disease after the second randomisation (Pathway 16 & 17), or after randomisation within the confirmatory trial testing the graduated biomarker signature-therapy combination (Pathway 18), all patients will discontinue and exit the ProBio trial.
